# CT-Based Radiomic Analysis for Preoperative Prediction of Tumor Invasiveness in Lung Adenocarcinoma Presenting as Pure Ground-Glass Nodule

**DOI:** 10.3390/cancers14235888

**Published:** 2022-11-29

**Authors:** Tzu-Ning Kao, Min-Shu Hsieh, Li-Wei Chen, Chi-Fu Jeffrey Yang, Ching-Chia Chuang, Xu-Heng Chiang, Yi-Chang Chen, Yi-Hsuan Lee, Hsao-Hsun Hsu, Chung-Ming Chen, Mong-Wei Lin, Jin-Shing Chen

**Affiliations:** 1 Department of Surgery, National Taiwan University Hospital and National Taiwan University College of Medicine, Taipei 100225, Taiwan; 2 Department of Pathology, National Taiwan University Hospital and National Taiwan University College of Medicine, Taipei 100225, Taiwan; 3 Institute of Biomedical Engineering, College of Medicine and College of Engineering, National Taiwan University, Taipei 106319, Taiwan; 4 Department of Thoracic Surgery, Massachusetts General Hospital, Boston, MA 02114, USA; 5 Institute of Epidemiology and Preventive Medicine, College of Public Health, National Taiwan University, Taipei 100025, Taiwan; 6 Department of Radiology, National Taiwan University Hospital and National Taiwan University College of Medicine, Taipei 100225, Taiwan; 7 Department of Surgical Oncology, National Taiwan University Cancer Center, No. 1, Sec. 1, Jen-Ai Rd., Taipei 106037, Taiwan

**Keywords:** ground-glass nodule, invasiveness, lung adenocarcinoma, lung cancer surgery, radiomic feature analysis

## Abstract

**Simple Summary:**

To forecast the invasiveness of the increasingly detected pure ground glass nodules, 338 cases were included in this study. Among them, 22.8% (77/338) of patients with pGGN were diagnosed with invasive adenocarcinoma. There were no nodal metastases or recurrence during a mean 78-month follow-up. A radiomic prediction model was constructed to predict the tumor’s invasiveness. The radiomic prediction model achieved good performance with an AUC of 0.7676. The prediction model can be used clinically in the treatment selection process.

**Abstract:**

It remains a challenge to preoperatively forecast whether lung pure ground-glass nodules (pGGNs) have invasive components. We aimed to construct a radiomic model using tumor characteristics to predict the histologic subtype associated with pGGNs. We retrospectively reviewed clinicopathologic features of pGGNs resected in 338 patients with lung adenocarcinoma between 2011–2016 at a single institution. A radiomic prediction model based on forward sequential selection and logistic regression was constructed to differentiate adenocarcinoma in situ (AIS)/minimally invasive adenocarcinoma (MIA) from invasive adenocarcinoma. The study cohort included 133 (39.4%), 128 (37.9%), and 77 (22.8%) patients with AIS, MIA, and invasive adenocarcinoma (acinar 55.8%, lepidic 33.8%, papillary 10.4%), respectively. The majority (83.7%) underwent sublobar resection. There were no nodal metastases or tumor recurrence during a mean follow-up period of 78 months. Three radiomic features—cluster shade, homogeneity, and run-length variance—were identified as predictors of histologic subtype and were selected to construct a prediction model to classify the AIS/MIA and invasive adenocarcinoma groups. The model achieved accuracy, sensitivity, specificity, and AUC of 70.6%, 75.0%, 70.0%, and 0.7676, respectively. Applying the developed radiomic feature model to predict the histologic subtypes of pGGNs observed on CT scans can help clinically in the treatment selection process.

## 1. Introduction

Lung cancer screening using computed tomography (CT) markedly reduces lung cancer mortality [1]. In recent years, an increasing number of early lung cancers have been detected via CT. One of the featuring imaging findings is the “ground-glass” appearance of a nodule (GGN) [2]. The histology associated with GGNs can be inflammation, interstitial fibrosis, atypical adenomatous hyperplasia, primary lung cancer, or even metastases. Multiple studies showed that the percentage of malignancy is lower in pure GGNs (pGGNs) than in solid nodules [3,4,5,6]. The general principle for the management of pGGNs is watchful waiting. For small or stable pGGNs, most guidelines suggest a 3-month to 1-year follow-up with CT scanning [7,8,9,10,11,12]. Biopsy or surgical resection is discussed only when an enlarged growth, especially solid parts, is detected [7,8,9,10]. Although benign lesions account for a large number of pGGNs, a certain percentage of invasive malignancy is still detected via surgical resection, varying from 1.7% to 24.3% [13,14,15].

The International Association for the Study of Lung Cancer, the American Thoracic Society, and the European Respiratory Society have already recognized the different prognoses of AIS, MIA, and invasive adenocarcinoma [16]. The experts devised the new AIS/MIA classifications because these groups of patients possess 100% or near 100% disease-free survival following complete resection, which was different from the more aggressive invasive adenocarcinoma.

Considering the favored treatment choice of pure or near pure (CT ratio < 25%) ground glass nodules, sublobar resection was widely recognized [17,18]. However, a different, more appropriate surgical method can be considered based on the pathology result. If the lesion is AIS/MIA, then a wide wedge resection without lymph node dissection is recommended [19]. However, according to the results of JCOG0802, segmentectomy would be preferred for peripheral lung invasive adenocarcinoma with a tumor size of less than 2 cm [20]. Therefore, the preoperative prediction of tumor invasiveness may help chest surgeons select the appropriate surgical method for early-stage lung adenocarcinoma.

Common parameters for predicting invasiveness include GGN size, CT Hounsfield unit (HU) scales, or the growth of GGN sizes within certain periods [4,13,14,15,21]. However, these conventional features alone cannot detect pathological invasiveness. The identification of additional features may help aid pGGN management. Radiomic feature analysis has emerged as a new method for pathological feature prediction [22]. It allows a more objective way of processing the large amount of information extracted from CT images. Several CT-based radiomic prediction models have been developed for the differential diagnosis of benign and malignant lung nodules [23], prediction of invasive pathological features, and clinical outcomes of lung cancer [24,25]. However, to date, only one previous study has applied radiomic feature analysis to predict the histologic subtype in a cohort comprising only pGGNs [26].

Currently, we lack detailed features and long-term clinical outcomes following surgery in a large cohort of pGGN lung adenocarcinoma. We aimed to analyze the clinicopathological characteristics and outcomes of surgically resected lung adenocarcinoma with pGGNs at a single institution and to construct a radiomic model using radiologic features to predict the histologic subtypes of pGGNs.

## 2. Materials and Methods

### 2.1. Study Population

**Figure 1:**
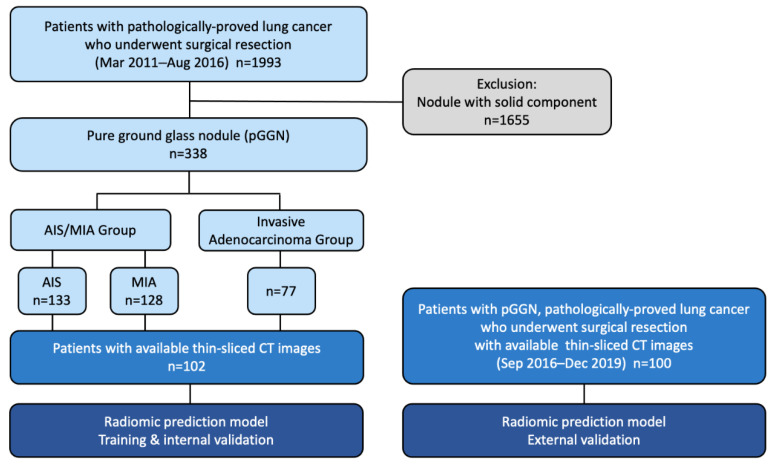
Flowchart of the study population.

From March 2011 to August 2016, 1993 consecutive lung cancer patients undergoing pGGN lung tumor resection via the same clinical protocols and perioperative care at National Taiwan University Hospital (NTUH) were reviewed retrospectively. Data were obtained from a prospectively collected database. Preoperative chest CT scans were reviewed by two qualified specialists (thoracic surgeons or thoracic radiologists) using a commercially available software viewer (IMPAX 5.2; Agfa HealthCare N.V., Mortsel, Belgium) independently. The ground glass part was defined by pulmonary attenuation with preservation of the bronchial and vascular margins, while solid parts obscured the background structures [27]. Consolidation-to-tumor ratio (C/T ratio) and the maximal solid part diameter to the maximal GGN diameter on the axial view were measured [3,4]. Cases with controversial or equivocal results were further reviewed by a senior thoracic surgeon (M.W.L) at a multidisciplinary conference. Patients with part- or pure-solid nodules on chest CT images were excluded. Finally, 338 patients with surgically resected pGGNs at the NTUH were enrolled. Another 100 cases from the same institution from September 2016 to December 2019 were enrolled retrospectively as well for radiomic model external validation (Figure 1). This retrospective study was approved by the research ethics committee of NTUH (project approval number: 201910065RINB, 202112105RINB, approval date: 15 November 2019; 17 February 2022), and the requirement for informed patient consent was waived. The clinical stages were determined according to the 8th American Joint Committee on Cancer (AJCC) staging for lung cancer [28]. The indications for resection of pGGNs via video-assisted thoracoscopic surgery (VATS) included large GGNs (>8 mm) and interval tumor growth noted on follow-up CT scans. For pGGNs smaller than 8 mm or with stationary size, tumor resection would be arranged after 6–12 months of follow-up due to the patient’s request out of personal will or lung cancer family history.

### 2.2. Pathological Data Review

The hematoxylin and eosin-stained permanent section slides were reviewed by two senior thoracic pathologists (M.S.H and Y.H.L) independently. Histologic classification and pathological features were classified according to the 2021 World Health Organization classification of thoracic tumors [29]. Histologic subtypes were classified into five categories (lepidic, acinar, papillary, micropapillary, and solid), with the percentages recorded [30]. We used the IASLC proposed grading system for invasive non-mucinous adenocarcinoma for tumor grading: lepidic-predominant case with no or no more than 20% of high-grade patterns as grade 1; acinar or papillary-predominant case with no or no more than 20% of high-grade patterns as grade 2; and those with more than 20%, as grade 3 [30]. Tumors spread through air spaces (STAS) implied tumor cells within the air spaces in the lung parenchyma at a distance of at least one alveolus away from the main tumor [31]. Based on the invasiveness of final pathology, we divided the cohort into two groups: AIS/MIA group, comprising patients with AIS and MIA, and invasive adenocarcinoma group, comprising those with invasive adenocarcinoma.

### 2.3. Radiomic Prediction Model Development

**Figure 2:**
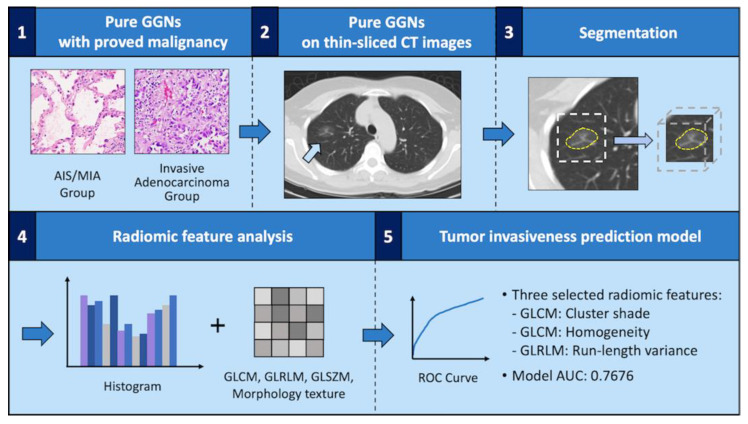
The framework of the tumor invasiveness prediction model for pure ground-glass nodules (pGGNs) lung adenocarcinoma. Summary of the radiomic analysis procedure: The steps involved in radiomics: (**1**) select patients with proven lung adenocarcinoma whose computed-tomography (CT) images were presented as pGGNs; (**2**) select patients with thin-sliced CT images; (**3**) segment tumor part in thin-sliced CT images; (**4**) extract radiomic information; (**5**) build a prediction model using extracted radiomic values and evaluate its performance.

The framework of the tumor invasiveness prediction model for pure ground-glass nodules (pGGNs) lung adenocarcinoma was demonstrated in Figure 2. The overall radiomic procedure was composed of (1) the pre-processing step, (2) the segmentation step, and (2) the characterizing step. For the pre-processing step, to minimize the impacts of spatial resolution, a normalization scheme for spatial resolution was performed by the trilinear interpolation using SciPy 1.4.1 (https://www.scipy.org/, accessed on 1 January 2022) in Python 3.6.3 (https://www.python.org/, accessed on 1 January 2022); the interpolation would resample each voxel into isotropic voxels, where one voxel corresponded to 1 mm. Furthermore, to maintain the original imaging phenotype of lesions, the intensity value of input data was consistent with the original Hounsfield Unit in CT. In the segmentation step, the border of the tumor was then extracted semi-automatically using an in-house segmentation method (Section 2.5). To separate the boundary from the surrounding normal tissue and fill to the missed area, the segmentation results were manually adjusted by two thoracic specialists (M-W Lin and X-H Chiang). In the characterizing step, the segmented lesion area was extracted with 404 radiomics features, including morphologic, histogram, and textural features (gray level co-occurrence matrix, GLCM; gray level run length matrix, GLRLM; gray level size zone matrix, GLSZM), which were then forwarded into the feature selection approach and applied to the selected features for model building (Supplementary Table S1). The features extraction was performed using Pyradiomics 2.2.0 (https://pyradiomics.readthedocs.io/en/latest/, accessed on 1 January 2022) in Python 3.6.3, and the details of quantization algorithms are available at https://pyradiomics.readthedocs.io/en/latest/features.html, accessed on 1 January 2022.

### 2.4. Image Acquisition

Pulmonary CT images were obtained using scanners from the following manufacturers: GE (LightSpeed Ultra, LightSpeed 16, LightSpeed VCT, and Discovery CT750 HD; GE, Chicago, IL, USA), Philips (iCT 256 and Ingenuity; Best, The Netherlands), Siemens (Definition AS+, Emotion 16, Sensation 16, Sensation 64; München, Germany), and Toshiba (Aquilion ONE; Tokyo, Japan). CT image parameters were as follows: 110–130 kVp; 10–758 mA; slice thickness, 0.625–1.25 mm; pixel spacing, 0.41–0.88 mm; and matrix, 512 × 512. The reconstruction kernel of CT images was determined using GE Standard kernel, Philip kernel B and L, Siemens kernel B40f and B50f, and Toshiba kernel FC08.

### 2.5. Segmentation

Semi-automatic segmentation was implemented using three-step processing, including: (1) pre-processing and volume-of-interest (VOI) extraction, (2) initial segmentation, and (3) post-processing for removing the attached vessel. In the first step, each voxel in the CT images was resampled into an isotropic voxel with a resolution of 1 mm using trilinear interpolation and SciPy 1.4.1 (https://www.scipy.org/, accessed on 1 January 2022) in Python 3.6.3 (https://www.python.org/, accessed on 1 January 2022). Subsequently, by manually selecting a voxel as the center of the lesion, a patch centered on this voxel with a 64 × 64 × 64 neighborhood was cropped as the VOI. In the second step, the hybrid level-set segmentation approach was applied for the initial segmentation [32]. The segmentation algorithm allowed the user to adjust the parameter μ, which was used to adjust the lower bound of the gray-level in the target lesion area, to make the segmentation algorithm handle varying CT attenuation types of lesions. Given that the surrounding vessel may present a similar intensity to the lesion, normal tissues would also be included in the initial segmented area. Thus, in the third step, a Frangi-based method was implemented to enhance the vessel area in the image, excluding the vessel by extracting the enhanced area [33]. Finally, the segmentation results were verified using two thoracic surgeons to ensure precise tumor boundaries. The segmentation algorithm was developed in MATLAB version 2018a (MathWorks, Natick, MA, USA).

### 2.6. Statistical Feature Extraction and Prediction Model Construction

The sequential forward selection (SFS) algorithm was applied to select significant features for model building. Before selection, all features were normalized by z-scores. Subsequently, the normalized features were forwarded into an iterative procedure of SFS. In the SFS, a feature that achieved the highest performance (i.e., accuracy) across the extracted features was selected first. Subsequently, from the remaining features, a feature that could further improve the performance in combination with the first selected feature was selected; the rest of the features were then selected as per this procedure until there was no further improvement in the performance. Based on the selected features, a logistic regression model based on a linear kernel was constructed. The feature selection and model construction were performed using the Statistics and Machine Learning Toolbox in MATLAB version 2018a (MathWorks, Natick, MA, USA).

To investigate if the proposed model could be used for tumor invasiveness prediction in the general setting, external validation was performed in an external validation cohort (*n* = 100) with lung cancer patients undergoing pGGN lung tumor resection.

### 2.7. Statistical Analyses

For the descriptive statistics of patient characteristics, pathological outcomes, and perioperative outcomes, number (percentage) is used for categorical variables, and mean ± standard deviation for continuous variables. Between the invasive adenocarcinoma and AIS/MIA groups, Student’s *t*-test was performed to compare continuous variables with normal distribution. Fisher’s exact test and Pearson’s chi-square test were used for categorical variables. Statistical significance was set at *p* < 0.05. The Kaplan–Meier survival curve was plotted for the disease progression-free survival analysis. The above statistical analysis was performed using IBM Statistical Product and Service Solutions (SPSS) Statistics for Mac (version 25.0; IBM Corp., Armonk, NY, USA).

Two-tailed Student’s *t*-tests were used to compare the extracted radiomic features between the invasive adenocarcinoma and AIS/MIA groups. The forward selection method was chosen to select the predictive features from the 404 radiomic features. Based on the selected feature set, a classification model was constructed using logistic regression. A leave-one-out cross-validation (LOOCV) procedure was implemented to evaluate the performance of the radiomic-based model. The prediction performance of the proposed model was evaluated using receiver operating characteristic curve (ROC)-area under curve (AUC) analysis, accuracy, sensitivity, and specificity; the cut-off value was determined by maximizing the Youden index. Furthermore, we implanted a predictive model from the study by Xu et al. [34] for patients with thin-slice CT (*N* = 102) to evaluate efficacy. Using the model built by the radiomic features proposed by them, ROC and AUC analyses were carried out. Statistical analysis was performed using the Statistics and Machine Learning Toolbox in MATLAB version 2018a (MathWorks, Natick, MA, USA). Statistical significance was set at *p* < 0.05.

## 3. Results

### 3.1. Patient Demographics and Clinicopathological Characteristics

Table 1 details patient demographics and clinical characteristics. The majority of patients were female (71.3%) and nonsmokers (92.6%). Their mean age was 55.9 years, mostly with fair performance status (82.0%). Over one-fourth of the patients (27.2%) had a family history of lung cancer. Higher serum carcinoembryonic antigen (CEA) levels were detected in seven patients only.

We divided the cohort into two groups according to the invasiveness of the final pathology: AIS/MIA versus invasive adenocarcinoma. Tumor diameters on the initial CT images and serum CEA levels were significantly larger in the invasive adenocarcinoma group. No other differences were observed between the groups.

### 3.2. Pathological Outcomes

**Figure 3:**
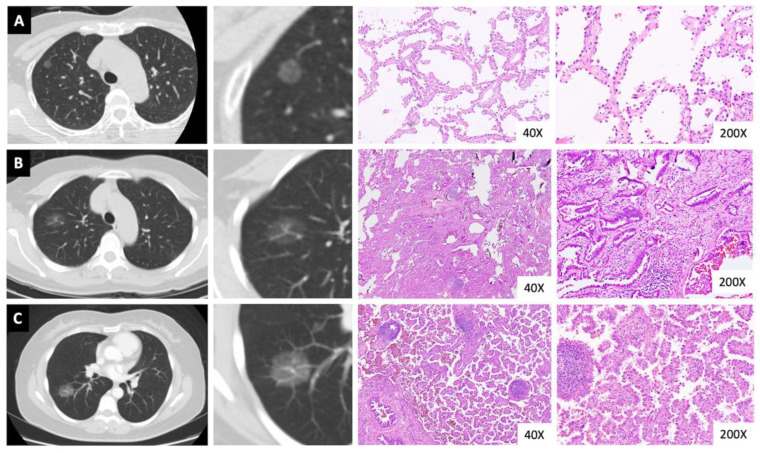
Computed tomography images with the corresponding microscopic pathology images. Represented cases: (**A**) adenocarcinoma in situ, (**B**) minimally invasive adenocarcinoma, and (**C**) papillary predominant invasive adenocarcinoma.

There were 133 (39.3%), 128 (37.9), and 77 (22.8%) patients diagnosed with lung AIS, MIA, and invasive adenocarcinoma, respectively. No lymphovascular invasion (LVI), visceral pleural invasion (VPI), STAS, lymph node metastases, or distant metastases were detected. According to the AJCC 8th lung cancer staging system, no patient had a stage exceeding stage IA. According to the IASLC grading system for invasive non-mucinous adenocarcinoma, all invasive adenocarcinoma cases were either of grade 1 (lepidic-predominant, 33.8%) or grade 2 (acinar, 55.8%; papillary, 10.4%), and none had more than 20% of high-grade patterns such as micropapillary, solid, or cribriform patterns. In this study, there were two acinar-predominant invasive adenocarcinomas possessing 10% and 15% micropapillary components, respectively. Details of the pathological outcomes are presented in Table 2, and the CT images with digital microscopic pathology images of AIS, MIA, and invasive adenocarcinoma in Figure 3.

### 3.3. Perioperative Outcomes and Survival

**Figure 4:**
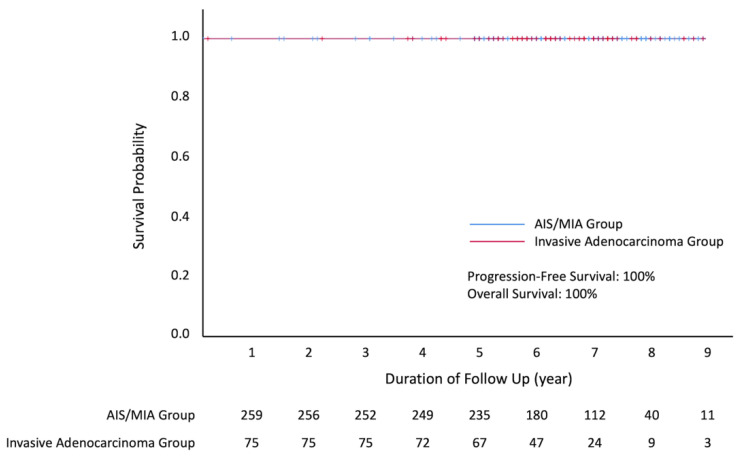
Kaplan–Meier curves of progression-free and overall survival for the adenocarcinoma in situ/minimally invasive adenocarcinoma group and invasive adenocarcinoma group.

All patients underwent VATS for tumor resection, mostly wedge resection (68.1%). About half of the patients had CT-guided localization, operated via a uniportal VATS setting and they underwent non-intubated surgery. Considering post-operative outcome, the median (interquartile range) of post-operative hospital stay was 3 (1) days. The surgical mortality rate within 30 days was 0%. The 5-year overall survival and 5-year progression-free survival were all 100%, with a follow-up time of 78 ± 18 months (mean ± standard deviation) (Figure 4). Details of perioperative outcomes are listed in Table 3.

### 3.4. Radiomic Feature Analysis

Comparison of partial radiomic features (26/404) revealed a significant difference (*p* < 0.05) between the AIS/MIA and invasive adenocarcinoma groups in some morphologic, histogram, GLCM, GLRLM, and GLSZM features. The details are listed in Table 4.

**Figure 5:**
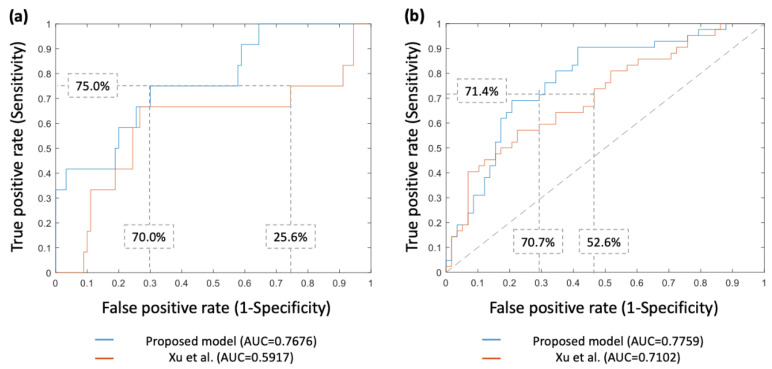
Receiver operating characteristics curves of tumor invasiveness prediction for pure ground-glass nodules (pGGNs) lung adenocarcinoma using the proposed and previously published models from Xu et al. [34] in (**a**) the internal validation (i.e., leave-one-out cross-validation) and (**b**) the external validation. ROC, receiver operating characteristic; AUC, area under the curve.

Based on forward selection, the cluster shade (GLCM), homogeneity (GLCM), and run-length variance (GLRLM) were selected as predictive factors to construct the prediction model. Using the selected features, the logistic regression model was built to classify the AIS/MIA and invasive adenocarcinoma groups, achieving accuracy, sensitivity, specificity, and AUC of 70.6%, 75.0%, 70.0%, and 0.7676, respectively. By implementing the predictive model proposed by Xu et al. [34], another ROC curve was plotted that revealed an AUC value of only 0.5917, which was lower than that of the prediction model we built (Figure 5a).

The clinicopathological features of the pGGN external validation cohort (*n* = 100) are listed in Table 5 and Table 6. For the external validation (Figure 5b), the proposed model achieved an accuracy, sensitivity, specificity, and AUC of 71.0%, 71.4%, 70.7%, and 0.7759, respectively; the model of Xu et al. [34] yielded AUC of 0.7102, which was lower than that of the proposed model. The performance was similar between the internal and external validation for the proposed model.

## 4. Discussion

Pure GGNs possess mostly benign characteristics [3,4]. Recommended management for pGGNs is watchful waiting [7,9,12]. Due to the excellent survival of pGGNs [5], serial investigations in the International Early Lung Cancer Action Project concluded that follow-up with only annual CT was safe until the growth of solid parts was detected [7,35]. Other guidelines also use different threshold sizes to determine surveillance protocols: 5 mm in the European Society of Thoracic Surgeons and the American College of Chest Physicians, and 6 mm in the Fleischner Society 2017 Guidelines, with intervals ranging from 3 months to 1 year [8,11]. The National Comprehensive Cancer Network also suggested a baseline annual low-dose CT surveillance for stable lesions smaller than 19 mm and scans at closer 6-month intervals for lesions larger than 19 mm; if the lesion sizes had increased by more than 1.5 mm within the follow-up period suggested, additional biopsy or resection was recommended [9]. Similarly, the British Thoracic Society guidelines advised that for lung nodules larger than 5 mm or those with a rapid size increase, CT surveillance should be performed in 3 months, with a further assessment based on related risks as needed [10].

The different prognoses of AIS, MIA, and invasive adenocarcinoma were widely recognized [16]. For AIS or MIA, a wide wedge without lymph node dissection would be sufficient [19], while a segmentectomy was more suitable for small peripheral invasive adenocarcinoma [20]. If the invasiveness of a pGGN could be predicted, then it certainly would help thoracic surgeons make a more precise and appropriate surgical decision. In our study, over one-fifth (22.78%) of the pGGNs were found to be invasive adenocarcinoma, which is consistent with the malignancy rate (1.7–24.3%) shown in previous studies [13,14,15]. These results suggest the possibility of underestimating cancer diagnosis and staging, suggesting a more definitive treatment modality at the time of discovery of a pGGN. Of note, in our cohort, all resected pGGNs harbored no characteristics associated with poor prognosis (e.g., LVI, VPI, and STAS); the recurrence rate in our cohort was 0%, and the 5-year overall and progression-free survival was 100%. Only two patients (0.6%) had tumors with micropapillary or solid components. Thus, although invasive adenocarcinoma was observed in over one-fifth of the patients with pGGNs, resection of the lesion appeared sufficient for cancer elimination.

To date, most studies predicted the invasiveness of lung pGGNs using conventional imaging parameters such as tumor sizes on CT scans, interval size changes, and mean HU values, with or without spiculation [4,13,14,15,16,17,18,19,20,21]. However, these features, alone or in combination, cannot accurately differentiate pathologic invasiveness. By converting imaging data into quantitative features via “feature extraction”, radiomic feature analysis has emerged as a more solid and objective strategy for processing a large amount of information on CT images, and further combination with machine learning strategies is also promising [34]. However, very few studies focused on pGGNs [26]. Although Xu et al. proposed a radiomic analysis to distinguish AIS/MIA from IA for pGGNs, their cohort showed a wide range of lesion density (−829.2 to −122.5 HU) [34]; their cohort may, thus, contain some part-solid nodules (density > −190 HU) [36] rather than full pGGNs. Accordingly, their methods may not be appropriate for a cohort without higher-density lesions. On applying their methods to our cohort, which lacks the higher density lesions (−817.1 to −612.8 HU, Table 1), a lower AUC value of 0.5917 was noted. Conversely, our methods that were based on the three features (i.e., the cluster shade [GLCM], homogeneity [GLCM], and Run-length variance [GLRLM]) for prediction achieved an overall AUC of 0.7676, showing better discrimination. This model can be used during the clinical decision-making process to anticipate whether a pGGN has invasive features and if it should be resected.

In this study, we estimated the cut-offs for sensitivity and specificity based on the clinical necessity of a sensitive diagnostic tool. Wedge resection is recommended for patients with AIS or MIA [18,19]. However, for patients with early-stage invasive adenocarcinoma, the standard surgical treatment is lobectomy [37]. According to the JCOG0802 trial published in 2022, segmentectomy may be the treatment of choice for early-staged peripheral invasive adenocarcinoma with a tumor size of less than 2 cm [20]. Therefore, a sensitive diagnostic tool should be used to preoperatively detect invasive histologic components for facilitating the decision of wedge resection for patients with AIS or MIA. Accordingly, we considered a method that could attain >75% sensitivity and maintain 70% specificity for this task. This detection tool may be used as an alternative to detect the invasive components. Specifically, if invasive adenocarcinoma was predicted, a surgeon could consider selecting lobectomy or segmentectomy to prevent malignant behaviors of invasive adenocarcinoma by undertreatment with wedge resection only.

This study has several limitations. First, given the small sample size, the cohort majorly comprising AIS/MIA, and invasive adenocarcinoma causing data imbalance, confident and safe clinical application of our model could be difficult and further validation would be required. Furthermore, a more balanced data set would be required to fit the proposed model and to prevent the model from predicting the major class (i.e., AIS/MIA). This single-center study could also have low generalizability, and further external validation using a multi-center cohort is necessary. Image acquisition protocols and CT scanners varied during the study period. The study cohort comprised exclusively Asian patients; thus, extrapolation and application of the findings and the model may be difficult in other patient populations. Third, because of the varying acquisition protocols, the impact of CT parameters on model prediction should be further investigated. Finally, the proposed model is not fully automated; it requires semi-automatic segmentation of nodules; a robust automatic segmentation method may be required to further reduce the interobserver difference before its application in clinical practice.

## 5. Conclusions

We utilized machine learning techniques to develop a radiomic feature model that predicts the histologic subtype associated with a pGGN as observed on a CT scan, which can be used clinically in the treatment selection process.

## Figures and Tables

**Table 1:** Demographic and clinical features.

Variables	All (*n* = 338)	AIS/MIA (*n* = 261)	Invasive Adenocarcinoma (*n* = 77)	*p*-Value
Age > 65 years old	67 (19.8)	51 (19.5)	16 (20.8)	0.224
Sex				0.405
Female	241 (71.3)	191 (73.2)	50 (64.9)	
Male	97 (28.7)	70 (26.8)	27 (35.1)	
ECOG				0.329
0	277 (82.0)	204 (78.2)	73 (94.8)	
1	61 (18.1)	57 (21.8)	4 (5.2)	
≥2	0 (0.0)	0 (0.0)	0 (0.0)	
FVC (%)	109.2 ± 13.0	110.0 ± 13.2	107.9 ± 12.5	0.939
FEV1 (%)	109.4 ± 14.8	109.7 ± 15.5	108.8 ± 13.8	0.308
Smoker	25 (7.4)	20 (7.7)	5 (6.5)	0.102
Family history of lung cancer	92 (27.2)	75 (28.7)	17 (22.1)	0.568
Comorbidities	129 (38.2)	95 (36.4)	34 (44.2)	0.314
Type 2 diabetes mellitus	17 (5.0)	12 (4.6)	5 (6.5)	
Hypertension	65 (19.2)	51 (19.5)	14 (18.2)	
Cardiac diseases	29 (8.6)	25 (9.6)	4 (5.2)	
End-stage renal disease	4 (1.2)	4 (1.5)	0 (0.0)	
History of other malignancies	53 (15.7)	48 (18.4)	5 (6.5)	
Abnormal serum CEA level	7 (2.1)	3 (1.2)	4 (5.2)	0.033
Thin-sliced CT images	102 (30.2)	90 (34.5)	12 (15.6)	0.002
Tumor size on CT images (cm)	1.1 ± 0.5	1.1 ± 0.4	1.3 ± 0.6	<0.001
Tumor density on CT images (HU)	−722.7 ± 47.3	−727.0 ± 46.3	−691.0 ± 45.1	0.013

Values are presented as *n* (%) or mean ± standard deviation. AIS, adenocarcinoma in situ; CEA, carcinoembryonic antigen; CT, computed tomography; ECOG, Eastern Cooperative Oncology Group; FVC, forced vital capacity; FEV1, forced expiratory volume in 1 s; HU, Hounsfield unit; MIA, minimally invasive adenocarcinoma.

**Table 2:** Pathological features.

Variables	All (*n* = 338)	AIS/MIA (*n* = 261)	Invasive Adenocarcinoma (*n* = 77)	*p*-Value
LVI	0 (0.0)	0 (0.0)	0 (0.0)	>0.999
VPI	0 (0.0)	0 (0.0)	0 (0.0)	>0.999
STAS	0 (0.0)	0 (0.0)	0 (0.0)	>0.999
Grading				<0.001
1	287 (84.9)	261 (100.0)	26 (33.8)	
2	51 (15.1)	0 (0.0)	51 (66.2)	
3	0 (0.0)	0 (0.0)	0 (0.0)	
Histological type				<0.001
AIS	133 (39.4)	133 (51.0)	0 (0.0)	
MIA	128 (37.9)	128 (49.0)	0 (0.0)	
IA	77 (22.8)	0 (0.0)	77 (100.0)	
Predominant subtype				<0.001
Lepidic	287 (84.9)	261 (100.0)	26 (33.8)	
Acinar	43 (12.7)	0 (0.0)	43 (55.8)	
Papillary	8 (2.4)	0 (0.0)	8 (10.4)	
Micropapillary	0 (0.0)	0 (0.0)	0 (0.0)	
Solid	0 (0.0)	0 (0.0)	0 (0.0)	
Presence of micropapillary orsolid components	2 (0.6)	0 (0.00)	2 (2.6)	0.009
T stage				<0.001
Tis	133 (39.4)	133 (51.0)	0 (0.0)	
T1mi	128 (37.9)	128 (49.0)	0 (0.0)	
T1a	42 (12.4)	0 (0.0)	42 (54.6)	
T1b	32 (9.5)	0 (0.0)	32 (41.6)	
T1c	6 (1.8)	0 (0.0)	6 (7.8)	
LN metastasis	0 (0.0)	0 (0.0)	0 (0.0)	>0.999
Distant metastasis	0 (0.0)	0 (0.0)	0 (0.0)	>0.999
TNM stage				<0.001
AIS	133 (39.4)	133 (51.0)	0 (0.0)	
IA1	170 (50.3)	128 (49.0)	42 (54.6)	
IA2	32 (9.5)	0 (0.0)	32 (41.6)	
IA3	6 (1.8)	0 (0.0)	6 (7.8)	

Values are presented as *n* (%) or mean ± standard deviation. LVI, lymphovascular invasion; STAS, spread through air spaces; VPI, Visceral pleural invasion. TNM staging, tumor, node, metastasis staging. See Table 1 legend for expansion of abbreviations.

**Table 3:** Perioperative and post-operative outcomes.

Variables	All (*n* = 338)	AIS/MIA (*n* = 261)	Invasive Adenocarcinoma (*n* = 77)	*p*-Value
Surgery method				<0.001
Wedge resection	230 (68.1)	187 (71.7)	43 (55.8)	
Segmentectomy	53 (15.7)	43 (16.5)	10 (13.0)	
Lobectomy	55 (16.3)	31 (11.9)	24 (31.2)	
Surgery approach				>0.999
VATS	338 (100.0)	261 (100.0)	77 (100.0)	
Thoracotomy	0	0	0	
Single Port VATS	169 (50.0)	133 (51.0)	36 (46.8)	0.401
Nonintubated VATS	160 (47.3)	130 (49.8)	30 (39.0)	0.886
Mean no. of dissected lymph node stations	3.0 (0–7)	2.9 (0–7)	3.4 (0–7)	0.043
Mean no. of dissected lymph nodes	7.2 (0–41)	6.9 (0–41)	8.3 (0–37)	0.050
Operation time (minute)	101.0 ± 40.5	99.2 ± 39.5	107.3 ± 43.3	0.079
Blood loss (mL)	4.3 (0–300)	4.5 (0–300)	3.8 (0–100)	0.623
Post-operative hospital stay (day)	3 (1)	3 (1)	4 (1)	0.077
ICU stay (day)	0 (0)	0 (0)	0 (0)	0.521
Chest tube drainage (day)	1.7 (0–16)	1.7 (0–11)	1.8 (0–16)	0.933
Morbidities				0.648
Prolonged air leak > 5 days	8 (2.4)	5 (1.9)	3 (3.9)	
Chylothorax	2 (0.6)	2 (0.8)	0	
Wound infection	1 (0.30)	1 (0.4)	0	
Hemothorax for re-open	0	0	0	
Vocal cord palsy	0	0	0	
30-day mortality	0	0	0	>0.999
Recurrence	0	0	0	>0.999

Values are presented as *n* (%), mean ± standard deviation, mean (range) or median (interquartile range). ICU, intensive care unit; IQR, interquartile range; VATS, video-assisted thoracoscopic surgery. See Table 1 legend for expansion of abbreviations.

**Table 4:** Radiomic features of pure ground-glass nodule adenocarcinoma.

	Mean ± Standard Deviation		*p*-Value
	Invasive Adenocarcinoma Group (*n* = 12)	AIS/MIA Group (*n* = 90)	
Morphological features			
Elongation	0.83 ± 0.07	0.81 ± 0.11	0.532
Flatness	0.67 ± 0.13	0.63 ± 0.12	0.363
MeshVolume	745.59 ± 901.85	556.76 ± 436.62	0.489
Sphericity	0.62 ± 0.08	0.59 ± 0.11	0.281
SurfaceArea	615.02 ± 521.77	576.76 ± 402.49	0.811
Histogram features			
Skewness	0.83 ± 0.32	1.04 ± 0.32	0.049
Kurtosis	2.85 ± 0.86	3.39 ± 1.04	0.064
Uniformity	0.003 ± 0.001	0.004 ± 0.001	<0.005
Entropy	5.87 ± 0.19	5.63 ± 0.22	<0.005
75th percentile (HU)	−615.33 ± 56.09	−667.44 ± 59.21	0.009
GLCM			
Autocorrelation	9648.29 ± 1620.72	8427.60 ± 1571.13	0.028
Contrast	136.19 ± 108.84	109.99 ± 87.14	0.438
Correlation	0.77 ± 0.11	0.74 ± 0.13	0.467
ClusterProminence	2,762,101.27 ± 1,823,084.98	1,684,816.56 ± 1,551,971.846	0.072
ClusterShade	4343.83 ± 8476.11	6282.64 ± 9819.01	0.477
MaximumProbability	0.004 ± 0.001	0.01 ± 0.00	<0.005
homogenity	0.11 ± 0.02	0.14 ± 0.03	<0.005
GLRLM			
ShortRunEmphasis	0.92 ± 0.02	0.91 ± 0.03	0.259
LongRunEmphasis	1.48 ± 0.17	1.57 ± 0.29	0.159
LowGrayLevelRunEmphasis	0.001 ± 0.00	0.002 ± 0.00	0.046
HighGrayLevelRunEmphasis	632.07 ± 97.21	554.47 ± 97.40	0.021
RunLengthVariance	0.00002 ± 0.00	0.0003 ± 0.00	<0.005
GLSZM			
SmallAreaEmphasis	0.42 ± 0.08	0.39 ± 0.13	0.213
LargeAreaEmphasis	327,774.4 ± 335,630.8	282,469.3 ± 440,822.3	0.679
LowGrayLevelZoneEmphasis	0.08 ± 0.02	0.09 ± 0.02	0.223
HighGrayLevelZoneEmphasis	18.01 ± 5.58	14.94 ± 4.37	0.090

AIS, adenocarcinoma in situ; MIA, minimally invasive adenocarcinoma.

**Table 5:** Demographic and clinical features of the additional pGGN cohort for external validation.

Variables	All (*n* = 100)	AIS/MIA (*n* = 58)	Invasive Adenocarcinoma (*n* = 42)	*p*-Value
Age > 65 years old	23 (23.0)	13 (22.4)	10 (23.8)	0.870
Sex				0.312
Female	72 (72.0)	44 (75.9)	28 (66.7)	
Male	28 (28.0)	14 (24.1)	14 (33.3)	
ECOG				0.123
0	83 (83.0)	51 (87.9)	32 (76.2)	
1	17 (17.0)	7 (12.1)	10 (23.8)	
≥2	0 (0.0)	0 (0.0)	0 (0.0)	
FVC (%)	108.5 ± 14.4	110.1 ± 14.3	106.3 ± 14.5	0.211
FEV1 (%)	107.9 ± 14.1	109.4 ± 13.9	105.8 ± 14.2	0.209
Smoker	7 (7.0)	3 (5.2)	4 (9.5)	0.400
Family history of lung cancer	24 (24.0)	14 (24.1)	10 (23.8)	0.970
Comorbidities	33 (33.0)	21 (36.2)	12 (28.6)	0.423
Type 2 diabetes mellitus	6 (6.0)	4 (6.9)	2 (4.8)	
Hypertension	16 (16.0)	9 (15.5)	7 (16.7)	
Cardiac diseases	8 (8.0)	4 (6.9)	4 (9.5)	
End-stage renal disease	0 (0.0)	0 (0.0)	0 (0.0)	
History of other malignancies	14 (14.0)	9 (15.5)	5 (11.9)	
Abnormal serum CEA level	2 (2.0)	1 (1.7)	1 (2.4)	0.909
Tumor size on CT images (cm)	1.9 ± 0.4	1.8 ± 0.4	2.0 ± 0.4	0.019
Tumor density on CT images (HU)	−717.1 ± 51.0	−731.1 ± 50.7	−697.8 ± 45.2	0.001

Values are presented as *n* (%) or mean ± standard deviation. AIS, adenocarcinoma in situ; CEA, carcinoembryonic antigen; CT, computed tomography; ECOG, Eastern Cooperative Oncology Group; FVC, forced vital capacity; FEV1, forced expiratory volume in 1 s; HU, Hounsfield unit; MIA, minimally invasive adenocarcinoma; C/T ratio, consolidation to tumor ratio.

**Table 6:** Pathological features of the additional pGGN cohort for external validation.

Variables	All (*n* = 100)	AIS/MIA (*n* = 58)	Invasive Adenocarcinoma (*n* = 42)	*p*-Value
LVI	3 (3.0)	0 (0.0)	3 (7.1)	0.039
VPI	1 (1.0)	0 (0.0)	1 (2.4)	0.238
STAS	7 (7.0)	0 (0.0)	7 (16.7)	0.001
Grading				<0.001
1	73 (73.0)	58 (100.0)	15 (35.7)	
2	27 (27.0)	0 (0.0)	27 (64.3)	
3	0 (0.0)	0 (0.0)	0 (0.0)	
Histological type				<0.001
AIS	24 (24.0)	24 (41.4)	0 (0.0)	
MIA	34 (34.0)	34 (58.6)	0 (0.0)	
IA	42 (42.0)	0 (0.0)	42 (100.0)	
Predominant subtype				<0.001
Lepidic	71 (71.0)	58 (100.0)	13 (31.0)	
Acinar	27 (27.0)	0 (0.0)	27 (64.3)	
Papillary	1 (1.0)	0 (0.0)	1 (2.4)	
Micropapillary	0 (0.0)	0 (0.0)	0 (0.0)	
Solid	0 (0.0)	0 (0.0)	0 (0.0)	
Presence of micropapillary orsolid components	0 (0.0)	0 (0.00)	0 (0.0)	>0.999
T stage				<0.001
Tis	24 (24.0)	24 (41.4)	0 (0.0)	
T1mi	34 (34.0)	34 (58.6)	0 (0.0)	
T1a	24 (24.0)	0 (0.0)	24 (57.1)	
T1b	17 (17.0)	0 (0.0)	17 (16.7)	
T1c	1 (1.0)	0 (0.0)	1 (2.4)	
LN metastasis	0 (0.0)	0 (0.0)	0 (0.0)	>0.999
Distant metastasis	0 (0.0)	0 (0.0)	0 (0.0)	>0.999
TNM stage				<0.001
AIS	24 (24.0)	24 (41.4)	0 (0.0)	
IA1	58 (58.0)	34 (58.6)	24 (57.1)	
IA2	17 (17.0)	0 (0.0)	17 (16.7)	
IA3	1 (1.0)	0 (0.0)	1 (2.4)	

Values are presented as *n* (%) or mean ± standard deviation. LVI, lymphovascular invasion; STAS, spread through air spaces; VPI, visceral pleural invasion. See Table 1 legend for expansion of abbreviations.

## Data Availability

All data generated or analyzed during this study are included in this published article.

## Supplementary Materials

The following supporting information can be downloaded at: https://www.mdpi.com/article/10.3390/cancers14235888/s1, Table S1: (A): Radiomics features used in this study; (B): Formula of radiomic features.
