# Supplementary material for: CT-Based Radiomic Analysis for Preoperative Prediction of Tumor Invasiveness in Lung Adenocarcinoma Presenting as Pure Ground-Glass Nodule
Source: Cancers (Basel). 2022 Nov 29;14(23):5888. doi: 10.3390/cancers14235888 (PMC9739513; doi:10.3390/cancers14235888)
Supplement: Supplementary file 1 [file cancers-14-05888-s001.zip › cancers-1869840-supplementary.pdf]

Supplementary Table S1A. Radiomics features used in this study

Morphology	First order statistic	Second order statistics		
Shape	Histogram	GLCM	GLRLM	GLSZM
Elongation	Mean	Autocorrelation	Short Run Emphasis	Small Zone Emphasis
Flatness	Variance	Contrast	Long Run Emphasis	Large Zone Emphasis
Least Axis Length	Standard Deviation	Correlation	Gray-Level Non-uniformity	Gray-Level Non-uniformity
Major Axis Length	Maximum	Cluster Prominence	Run-Length Non-uniformity	Zone-Size Non-uniformity
Maximum 2D Diameter Column	Minimum	Cluster Shade	Run Percentage	Zone Percentage
Maximum 2D Diameter Row	Skewness	Dissimilarity	Low Gray-Level Run Emphasis	Low Gray-Level Zone Emphasis
Maximum 2D Diameter Slice	Kurtosis	Energy	High Gray-Level Run Emphasis	High Gray-Level Zone Emphasis
Maximum 3D Diameter	2.5th Percentile	Entropy	Short Run Low Gray-Level Emphasis	Small Zone Low Gray-Level Emphasis
Mesh Volume	25th Percentile	Homogeneity	Short Run High Gray-Level Emphasis	Small Zone High Gray-Level Emphasis
Minor Axis Length	50th Percentile	Maximum probability	Long Run Low Gray-Level Emphasis	Large Zone Low Gray-Level Emphasis
Sphericity	75th Percentile	Sum of squares: Variance	Long Run High Gray-Level Emphasis	Large Zone High Gray-Level Emphasis
Surface Area	97.5th Percentile	Sum average	Gray-Level Variance	Gray-Level Variance
Surface Volume Ratio	Uniformity	Sum variance	Run-Length Variance	Zone-Size Variance
Voxel Volume	Entropy	Sum entropy<br>Difference variance		

		Difference entropy
		Information measure of correlation 1
		Information measure of correlation 2
		INV
		INN
		Inverse difference moment normalized

GLCM, gray-level co-occurrence matrix; GLRLM, gray-level run length matrix; GLSZM, gray-level size zone matrix; INV, inverse difference; INN, inverse difference normalized

Supplementary Table S1B. Formula of radiomic features

Feature	Formula
**Morphology (Shape)**	
**λmajor**, **λminor**, **λleast** are the length of the largest, second largest and smallest principal component axes; Nv represent the number of voxels included in the ROI; Nf represent the number of faces (triangles) defining the Mesh, for each face i in the mesh, defined by points ai,bi and ci, the (signed) volume Vf of the tetrahedron defined by that face and the origin of the image (O) is calculated; V the volume of the mesh in mm^3, calculated by mesh volume; A the surface area of the mesh in mm^2, calculated by surface area;	
Elongation	$\text{Elongation} = \frac{\sqrt{\lambda\text{minor}}}{\sqrt{\lambda\text{major}}}$
Flatness	$\text{Flatness} = \frac{\sqrt{\lambda\text{least}}}{\sqrt{\lambda\text{major}}}$
Least Axis Length	$\text{Least Axis Length} = 4\sqrt{\lambda least}$
Major Axis Length	$\text{Minor Axis Length} = 4\sqrt{\lambda minor}$
Maximum 2D Diameter Column	Largest pairwise Euclidean distance between tumor surface mesh vertices in the row-column (generally the axial) plane.
Maximum 2D Diameter Row	Largest pairwise Euclidean distance between tumor surface mesh vertices in the column-slice (generally the axial) plane.
Maximum 2D Diameter Slice	Largest pairwise Euclidean distance between tumor surface mesh vertices in the row-column (generally the axial) plane.
Maximum 3D Diameter	Largest pairwise Euclidean distance between tumor surface mesh vertices.
Mesh Volume(V)	$V = \sum_{i=1}^{Nf} \frac{Oai \cdot (Obi \times Oci)}{6}$
Minor Axis Length	$\text{Minor Axis Length} = 4\sqrt{\lambda minor}$

Sphericity	$\frac{\sqrt[3]{36\pi V^2}}{A}$
Surface Area(A)	$A = \sum_{i=1}^{Nf} \frac{1}{2} \lvert a_i b_i \times a_i c_i \rvert$
Surface Volume Ratio (SVR)	$\mathrm{SVR} = \frac{A}{V}$
Voxel Volume(V_{voxel})	$V_{voxel} = \sum_{k=1}^{Nv} V_k$
**First order statistics (Histogram)**	
Let X(i) and P(i) denote the grey level and density of the ith element in an image matrix that has N voxels, respectively.	
Mean	$Mean = \frac{1}{N}\sum_{i=1}^{N} \mathrm{X(i)}$
Variance (Var)	$Var = \frac{1}{N}\sum_{i=1}^{N} \mathrm{X(i)} - \bar{\mathrm{X}}$
Standard deviation (SD)	$SD = \frac{1}{N}\sum_{i=1}^{N} (\mathrm{X(i)} - \bar{\mathrm{X}})^2$
Maximum<br>Minimum	$Maximum = \max(\mathrm{X})$<br>$Minimum = \min(\mathrm{X})$
Uniformity	$Uniformity = \sum_{i=1}^{N_l} \mathrm{P}(i)^2$
Entropy	$Entropy = -\sum_{i=1}^{N_l} \mathrm{P}(i) \log_2 \mathrm{P}(i)$
Kurtosis	$Kurtosis = \frac{\frac{1}{\mathrm{N}}\sum_{\mathrm{i=1}}^{\mathrm{N}}(\mathrm{X(i)} - \bar{\mathrm{X}})^4}{(\sqrt{\frac{1}{\mathrm{N}}\sum_{\mathrm{i=1}}^{\mathrm{N}}(\mathrm{X(i)} - \bar{\mathrm{X}})^2})^4}$

Skewness	$$Skewness = \frac{\frac{1}{N}\sum_{i=1}^{N}(X(i)-\overline{X})^3}{(\sqrt{\frac{1}{N}\sum_{i=1}^{N}(X(i)-\overline{X})^2})^3}$$

Second order statistics

Grey-level co-occurrence matrix [1]

$\boldsymbol{P(i,j)}$ is the co-occurrence matrix, N_g is the number of discrete intensity levels in the image, μ is the mean of $\boldsymbol{P(i,j)}$, $\mu_x(i)$ is the mean of $P_x(i)$, $\mu_y(j)$ is the mean of $P_y(j)$, $\sigma_x(i)$ is the standard deviation of $P_x(i)$, and $\sigma_y(j)$ is the standard deviation of $P_y(j)$, $P_{x+y}(k)$ is $\sum_{i=1}^{N_g}\sum_{j=1}^{N_g}\boldsymbol{P(i,j)}$ where $k = i + j$, $P_{x-y}(k)$ is $\sum_{i=1}^{N_g}\sum_{j=1}^{N_g}\boldsymbol{P(i,j)}$ where $k = |i - j|$, HX is the entropy of $P_x(i)$, HY is the entropy of $P_y(j)$, HXY is the entropy of $P(i,j)$, HXY1 is $\sum_{i=1}^{N_g}\sum_{j=1}^{N_g}\boldsymbol{P(i,j)\log_2(P_x(i)P_y(j))}$, HXY2 is $\sum_{i=1}^{N_g}\sum_{j=1}^{N_g}\boldsymbol{P_x(i)P_y(j)\log_2(P_x(i)P_y(j))}$, $\boldsymbol{DA}$ is the difference average .

Autocorrelation	$$Autocorrelation = \sum_{i=1}^{N_g}\sum_{j=1}^{N_g} ijP(i,j)$$
Contrast	$$Contrast = \sum_{i=1}^{N_g}\sum_{j=1}^{N_g}\lvert i-j\rvert^2 P(i,j)$$
Correlation	$$Correlation = \frac{\sum_{i=1}^{Ng}\sum_{j=1}^{Ng} ijP(i,j) - \mu_i(i)\mu_i(j)}{\sigma_x(i)\sigma_y(j)}$$
Cluster Prominence (CP)	$$CP = \sum_{i=1}^{Ng}\sum_{j=1}^{Ng}(i+j-\mu_i(i)-\mu_i(j))^4 P(i,j)$$
Cluster Shade (CS)	$$CS = \sum_{i=1}^{Ng}\sum_{j=1}^{Ng}(i+j-\mu_i(i)-\mu_i(j))^3 P(i,j)$$
Dissimilarity	$$Dissimilarity = \sum_{i=1}^{N_g}\sum_{j=1}^{N_g}\lvert i-j\rvert P(i,j)$$
Energy	$$Energy = \sum_{i=1}^{Ng}\sum_{j=1}^{Ng} P(i,j)^2$$

Entropy	$Entropy = -\sum_{i=1}^{Ng}\sum_{j=1}^{Ng} P(i,j) log_2 P(i,j)$
Homogeneity	$Homogeneity = \sum_{i=1}^{N_g}\sum_{j=1}^{N_g} \frac{P(i,j)}{1+\lvert i-j\rvert^2}$
Maximum probability	$Maximum\ probability = \max(P(\mathrm{i},\mathrm{j}))$
Sum of squares: Variance	$Variance = \sum_{i=1}^{N_g}\sum_{j=1}^{N_g} (i-\mu_x(i))^2 P(i,j)$
Sum average (SA)	$SA = \sum_{k=2}^{2N_g} P_{x+y}(k)k$
Sum variance	$Sum\ variance = \sum_{k=2}^{2N_g} (k-SA)P_{x+y}(k)$
Sum entropy	$Sum\ entropy = -\sum_{k=2}^{2N_g} P_{x+y}(k)\log_2(P_{x+y}(k))$
Difference variance	$Difference\ variance = \sum_{k=0}^{N_g-1} (k-DA)P_{x-y}(k)$
Difference entropy	$Difference\ entropy = -\sum_{k=0}^{N_g-1} P_{x-y}(k)\log_2(P_{x-y}(k))$
Information measure of correlation 1 (IMC1)	$IMC1 = \mathrm{HXY} - \frac{\mathrm{HXY1}}{\max\{HX, HY\}}$
Information measure of correlation 2 (IMC2)	$IMC2 = \sqrt{1-e^{-2(HXY2-HXY)}}$
Inverse difference (INV)	$INV = \sum_{k=0}^{Ng-1} \frac{P_{x-y}(k)}{1+\ k}$

Inverse difference normalized (INN)	$$INN = \sum_{k=0}^{Ng-1} \frac{P_{x-y}(k)}{1+\ k/Ng}$$
Inverse difference moment normalized (IDMN)	$$IDMN = \sum_{k=0}^{Ng-1} \frac{P_{x-y}(k)}{1+\ (k/Ng)^2}$$

Grey-level run length matrix [2]

$\boldsymbol{P}(\mathbf{i},\mathbf{j})$ is the run length matrix, N_g is the number of gray level in the image, N_r is the number of different run lengths, N_p is the number of voxels in the image, $\boldsymbol{\mu_0}$ is $\sum_{i=1}^{N_g}\sum_{j=1}^{N_r}\boldsymbol{P(i,j)i/\mathrm{N}_r}$, $\boldsymbol{\mu_r}$ is $\sum_{i=1}^{N_g}\sum_{j=1}^{N_r}\boldsymbol{P(i,j)j/\mathrm{N}_r}$.

Short Run Emphasis (SRE)	$$SRE = \frac{\sum_{i=1}^{N_g}\sum_{j=1}^{N_r}\frac{P(i,j)}{j^2}}{\mathrm{N}_r}$$
Long Run Emphasis (LRE)	$$LRE = \frac{\sum_{i=1}^{N_g}\sum_{j=1}^{N_r} j^2 P(i,j)}{\mathrm{N}_r}$$
Gray-Level Non-uniformity (GLN)	$$GLN = \frac{\sum_{i=1}^{N_g}(\sum_{j=1}^{N_r} P(i,j))^2}{\mathrm{N}_r}$$
Run-Length Non-uniformity (RLN)	$$RLN = \frac{\sum_{j=1}^{N_r}(\sum_{i=1}^{N_g} P(i,j))^2}{\mathrm{N}_r}$$
Run Percentage (RP)	$$RP = \frac{\mathrm{N}_r}{\mathrm{N_p}}$$
Low Gray-Level Run Emphasis (LGRE)	$$LGRE = \frac{\sum_{i=1}^{N_g}\sum_{j=1}^{N_r}\frac{P(i,j)}{i^2}}{\mathrm{N}_r}$$
High Gray-Level Run Emphasis (HGRE)	$$HGRE = \frac{\sum_{i=1}^{N_g}\sum_{j=1}^{N_r} i^2 P(i,j)}{\mathrm{N}_r}$$
Short Run Low Gray-Level Emphasis (SRLGE)	$$SRLGE = \frac{\sum_{i=1}^{N_g}\sum_{j=1}^{N_r}\frac{P(i,j)}{i^2 j^2}}{\mathrm{N}_r}$$

Gray level size zone matrix [3]

$P(\mathrm{i},\mathrm{j})$ is the size zone matrix, N_g is the number of gray level in the image, N_z is the number of different size zones, N_p is the number of voxels in the

**image,** $\boldsymbol{\mu_0}$ **is** $\sum_{i=1}^{N_g}\sum_{j=1}^{N_z}\boldsymbol{P(i,j)i/\mathrm{N}_z}$**,** $\boldsymbol{\mu_z}$ **is** $\sum_{i=1}^{N_g}\sum_{j=1}^{N_z}\boldsymbol{P(i,j)j/\mathrm{N}_z}$**.**	
Small Zone Emphasis (SZE)	$$SZE = \frac{\sum_{i=1}^{N_g}\sum_{j=1}^{N_z}\frac{P(i,j)}{j^2}}{\mathrm{N}_z}$$
Large Zone Emphasis (LZE)	$$LZE = \frac{\sum_{i=1}^{N_g}\sum_{j=1}^{N_z}j^2 P(i,j)}{\mathrm{N}_z}$$
Gray-Level Non-uniformity (GLN)	$$GLN = \frac{\sum_{i=1}^{N_g}(\sum_{j=1}^{N_z}P(i,j))^2}{\mathrm{N}_z}$$
Zone-Size Non-uniformity (ZLN)	$$ZLN = \frac{\sum_{j=1}^{N_z}(\sum_{i=1}^{N_g}P(i,j))^2}{\mathrm{N}_z}$$
Zone Percentage (ZP)	$$ZP = \frac{\mathrm{N}_z}{\mathrm{N}_\mathrm{p}}$$
Low Gray-Level Zone Emphasis (LGZE)	$$LGZE = \frac{\sum_{i=1}^{N_g}\sum_{j=1}^{N_z}\frac{P(i,j)}{i^2}}{\mathrm{N}_z}$$
High Gray-Level Zone Emphasis (HGZE)	$$HGZE = \frac{\sum_{i=1}^{N_g}\sum_{j=1}^{N_z}i^2 P(i,j)}{\mathrm{N}_z}$$
Small Zone Low Gray-Level Emphasis (SZLGE)	$$SZLGE = \frac{\sum_{i=1}^{N_g}\sum_{j=1}^{N_z}\frac{P(i,j)}{i^2 j^2}}{\mathrm{N}_z}$$
Small Zone High Gray-Level Emphasis (SZHGE)	$$SZHGE = \frac{\sum_{i=1}^{N_g}\sum_{j=1}^{N_r}\frac{i^2 P(i,j)}{j^2}}{\mathrm{N}_z}$$
Large Zone Low Gray-Level Emphasis (LZLGE)	$$LZLGE = \frac{\sum_{i=1}^{N_g}\sum_{j=1}^{N_z}\frac{j^2 P(i,j)}{i^2}}{\mathrm{N}_z}$$
Large Zone High Gray-Level Emphasis (LZHGE)	$$LZHGE = \frac{\sum_{i=1}^{N_g}\sum_{j=1}^{N_z}i^2 j^2 P(i,j)}{\mathrm{N}_z}$$
Gray-Level Variance (GLV)	$$GLV = \frac{\sum_{i=1}^{N_g}\sum_{j=1}^{N_z}P(i,j)(i-\mu_0)^2}{\sum_{i=1}^{N_0}\sum_{j=1}^{N_z}P(i,j)}$$

Zone-Size Variance (ZLV)	$ZLV = \frac{\sum_{i=1}^{N_g}\sum_{j=1}^{N_z} P(i,j)(j-\mu_z)^2}{\sum_{i=1}^{N_0}\sum_{j=1}^{N_z} P(i,j)}$
